# Scientific preparation for JRT: Wind pressure prediction model for large radio telescope based on real data from multi-sensors

**DOI:** 10.1016/j.heliyon.2024.e37892

**Published:** 2024-09-14

**Authors:** Rui Wu, Zhong Cao, Feng Wang, Rui Rao, Yuxiang Huang, Ruifeng Hu

**Affiliations:** a School of Electronics and Communication Engineering, Guangzhou University, Guangzhou 510006, China; b Center For Astrophysics, Guangzhou University, Guangzhou 510006, China; c Great Bay Center, National Astronomical Data Center, Guangzhou, Guangdong 510006, China; d Peng Cheng Laboratory, Shenzhen 518000, China; e Research Center for Wind Engineering and Engineering Vibration, Guangzhou University, Guangzhou 510006, China; f Yunnan Observatory, Chinese Academy of Sciences, Kunming 650011, China

**Keywords:** Wind pressure prediction, Large radio telescope, Variational mode decomposition, Pointing accuracy, Multi-sensors

## Abstract

Jingdong 120-meter radio telescope (JRT) is poised to become the world's largest single-aperture fully steerable medium-low frequency radio telescope. However, like other large-aperture radio telescopes, the JRT is vulnerable to wind loads, which can cause structural deformation and pointing errors. Addressing this challenge requires the ability to predict dynamic winds in real-time. This study developed a wind pressure preprocessing and prediction model using sensor data collected from the Kunming 40-meter radio telescope (KRT), enabling real-time prediction of wind pressure on the telescope. The model employs adaptive noise and Variational Mode Decomposition (VMD) techniques to eliminate random noise from the original wind pressure data. Subsequently, wind pressure predictions are made using a Bidirectional Long Short-term Memory (BiLSTM) model. By conducting predictions under various stabilization conditions and conducting a thorough analysis of measurement data from five sensors, the study has achieved impressive results in predicting wind pressure on the KRT reflector surface. The proposed model demonstrates the lowest MAE, RMSE, and MAPE, while achieving the highest $R^{2}$ across various data sets. Where the average $R^{2}$ of the proposed model is 0.9392 at 45° pitch angle attitude and the RMSE, MAE and MAPE values are 1.4923, 1.2377 and 1.82% respectively. This model helps wind load monitoring of real-time wind pressure monitoring of the telescope surface, to study the effects of wind load on pointing accuracy. By adjusting the control parameters to reduce wind load interference, to ensure the high-precision work of a large radio telescope, such as JRT.

## 1 Introduction

Radio telescopes facilitate astronomical observation by detecting and receiving radio waves, playing a pivotal role in various scientific endeavors such as satellite communication, manned spaceflight, and space exploration. They are instrumental in advancing the field of radio astronomy, contributing significantly to our understanding of the universe. Establishing astronomical observatories is a challenging task, as less than 3% of the Earth's surface is deemed optimal for this purpose. Factors such as wind speed serve as crucial considerations in selecting suitable locations for observatories [1]. Wind loading represents a significant challenge for telescopes, as it can lead to surface deformation and pointing errors. Control and pointing issues often arise during the design, testing, and operation of antennas, radio telescopes, and optical telescopes, with wind being a primary source of disturbance [2].

The Jingdong 120-meter pulsar radio telescope (JRT), slated to be constructed in Jingdong, Yunnan Province, is poised to become the world's largest medium and low-frequency fully steerable pulsar radio telescope. The core scientific objectives of JRT are the construction of a pulsar time reference system and the detection of nanohertz low-frequency gravitational waves [3]. At the same time, it also serves the pulse industry's National strategic needs in the fields of satellite navigation, deep space exploration, and space target radar monitoring.

As the antenna aperture of large radio telescopes increases, so does the wind-receiving area of the antenna reflector surface, consequently amplifying the wind load endured by the antenna [4]. This heightened wind load poses significant challenges, particularly as the overall performance demands of the antenna escalate. Consequently, the requirements for telescope pointing accuracy have intensified. However, the ramifications of on-site wind disturbance on the efficiency and pointing accuracy of large antennas have become increasingly pronounced.

The main methods used to study the wind load characteristics of radio telescope antenna structures encompass wind tunnel tests, numerical simulations, and field measurements. Wind tunnel experiments are instrumental in analyzing the wind pressure distribution on the main reflector for stress state analysis in large aperture radio telescopes [5]. Alexandrou et al. utilized wind tunnel tests to evaluate the wind loads on an 8-meter class astronomical telescope designed for open-air operation, determining both static and dynamic wind loads on the telescope structure and primary mirror [6]. Similarly, Vogiatzis et al. outlined a strategy for simulating wind load effects on large telescopes like the Thirty Meter Telescope (TMT) using computational fluid dynamics [7]. Numerical simulation methods are also widely employed to assess wind load characteristics. For example, He et al. utilized numerical simulations to calculate and analyze wind pressure distribution and wind force coefficients of different reflector groups with varying upwind postures for the Xinjiang Qitai 110-meter radio Telescope (QTT) [8]. Li et al. used numerical simulations to determine wind pressure distribution for different reflector groups with varying upwind postures [9]. Field measurements provide crucial real-world data for understanding wind load effects. Researchers have analyzed wind environment data and wind pressure data from various telescopes such as the Telescopio Nazionale Galileo (TNG), Carlsberg Automatic Meridian Circle (CAMC), and Nordic Optical Telescope (NOT), including parameters like wind speed, wind direction, average, and pulsating wind pressure, to assess their impact on observation accuracy [10]. Wang et al. established a parametric finite element model of a radio telescope to simulate its dynamic response under wind load excitation [11].

Bolbasova et al. conducted a seasonal study of wind speed vertical distribution and daytime optical turbulence conditions at the Baikal Astrophysical Observatory [12]. Additionally, research conducted by Dhananjay employed in situ evaluations using microthermal measurements for the Indian National Large Solar Telescope [13]. While wind tunnel experiments and numerical simulations provide valuable insights, they are often constrained by high costs, model assumptions, numerical errors, and computational time. Conversely, field measurements offer real-time data capturing, facilitating quicker identification of potential issues or optimization points, thereby enhancing telescope performance and stability.

According to reports on the Green Bank Telescope (GBT) [14], a 100-meter aperture fully steerable radio telescope, its antenna pointing accuracy can reach up to 1.5” at wind speeds below 2.5 m/s. However, as wind speeds increase, the pointing error caused by wind disturbance becomes increasingly detrimental to observations. For instance, at wind speeds exceeding 3 m/s, observations above 40 GHz are affected, and wind speeds surpassing 5 m/s limit observations above 20 GHz. Furthermore, if wind speeds persist above 11 m/s, all observations must cease. The distribution pattern of wind coefficients and wind pressures is influenced by multiple factors, resulting in complex causation. Therefore, understanding the flow characteristics of large radio telescope structures in wind fields and discerning wind pressure distribution patterns on large reflector surfaces are crucial for mitigating the impact on antenna efficiency and pointing accuracy.

With the emergence of Artificial Intelligence (AI) and Machine Learning (ML), driven by enhanced computing power and sophisticated databases, significant strides have been made. ML algorithms have advanced to the point where they can address the three “non-” issues in wind engineering: non-stationarity, non-Gaussianity, and non-linearity [15]. Given its capability to elucidate complex, non-linear relationships between inputs and outputs, ML emerges as a promising alternative to traditional methods such as wind tunnel experiments and Computational Fluid Dynamics (CFD) simulations in predicting wind pressure on structures [16]. Wei et al. used sensors installed on the physical telescope to collect real-time local data. They then created digital telescope models using this sensor data. These models simulated and calculated the global state of the telescope based on the service unit and predicted the behaviors and rules of the physical telescope using the generated twin data [17].

Deep Learning (DL) techniques have emerged as powerful tools in structural wind engineering research, demonstrating successful implementation across various domains. These applications include predicting wind-induced pressure time series, overall loads, aeroelastic responses, and wind gust estimates [18]. For instance, Artificial Neural Networks (ANN) have been utilized to forecast wind pressure coefficients for different roof sizes and slopes, considering building geometry and wind attack angles [19]. ANN models have also been employed to estimate pressure coefficients on building walls and roofs [20] and predict mean and Root-Mean-Square (RMS) pressure coefficients on gable roofs of low buildings [21]. Backpropagation Neural Networks (BPNN) and Fuzzy Neural Networks (FNN) have been developed to predict mean, RMS pressure coefficients, and time series of wind-induced pressures on large structures such as gymnasium roofs [22]. Additionally, BPNN combined with Proper Orthogonal Decomposition (POD-BPNN) has been introduced for predicting mean, RMS pressure coefficients, and time series of wind-induced pressures on building surfaces [23]. A novel approach combining Long Short-Term Memory network (LSTM) with POD has been proposed for predicting wind pressure time series on structures [24]. Furthermore, an improved neural network technique called Wavelet Neural Network (WNN) has been introduced to forecast pressure time series on high-rise structures, outperforming BPNN and Genetic Algorithm BPNN (GA-BP) [25].

Accurately estimating wind pressure on the reflector surface of radio telescopes is crucial for reflecting their load distribution characteristics accurately. Inaccurate predictions can lead to incorrect calculations of reflector deformation, failing to capture the true impact of wind. Even minor errors in wind pressure estimation can affect data precision. Therefore, accurately modeling wind pressure and deploying sensors for real-time data are essential to protect equipment and ensure data reliability.

**Figure 1:**
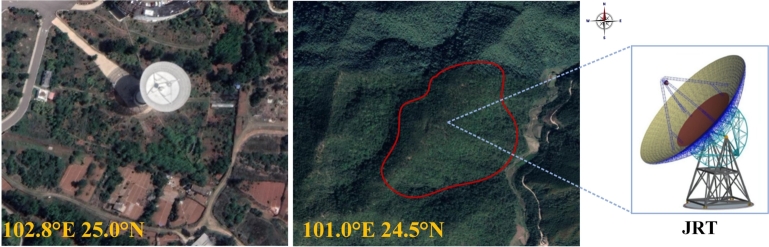
Geographic location of KRT site and JRT site.

**Figure 2:**
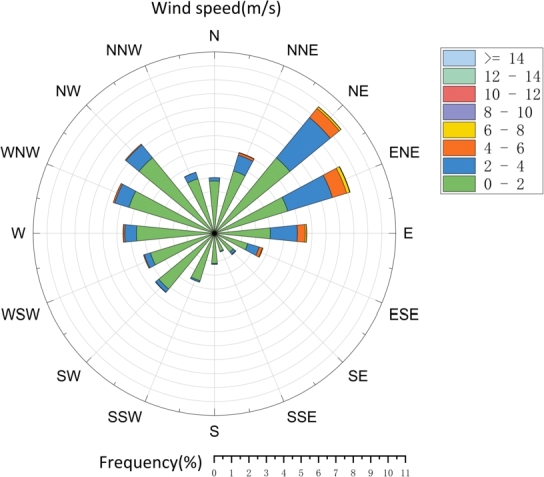
Wind rose diagram.

Since a substantial body of research suggests that standalone models often fail to deliver satisfactory results, recent studies have shifted their focus towards hybrid models that promise enhanced prediction accuracy. Notably, the incorporation of signal decomposition techniques and advanced forecasting methods has been shown to significantly enhance predictive performance by mitigating the nonlinearities and instabilities inherent in the original time series data [26]. Currently, common signal decomposition methods include Wavelet Transform (WT), Empirical Mode Decomposition (EMD), and Variational Mode Decomposition (VMD), among others. A one-dimensional Convolutional Neural Network based on Empirical Mode Decomposition (EMD-1DCNN) was applied to predict single, short-term, and long-term wind pressures by [27]. Non-Gaussian unsteady wind pressure prediction is successfully realized by [28]. Additionally, methods for predicting wind speed in advance near the QTT site are available. These methods employ modal decomposition combined with time-series prediction models to forecast wind conditions, thus enabling proactive measures [29]. Moreover, a hybrid decomposition algorithm, based on Wavelet Packet Decomposition (WPD) and Variational Mode Decomposition (VMD), has been proposed for predicting wind pressure on structures [30]. Upon comparison, it becomes evident that the signal denoising ability of wavelet decomposition depends on the degree of decomposition and the choice of mother wavelet. Meanwhile, EMD also suffers from shortcomings such as the endpoint effect and modal aliasing, which may limit its effectiveness in decomposition. In contrast, VMD demonstrates superior adaptive ability, capable of eliminating random fluctuations and addressing its own shortcomings. The Kunming 40-meter radio telescope (KRT) is a Very Long Baseline Interferometry (VLBI) facility in China, situated on Fenghuang Mountain, approximately 10 kilometers east of Kunming city [31]. The geographical features of the KRT location closely resemble those of the JRT, as both are situated in Yunnan Province. The Geographic location of KRT site and JRT site are shown in Fig. 1. Meteorological data on wind speed and direction over the past year were systematically collected using a meteorological tower at the KRT site. The resulting wind rose diagrams, encapsulated in Fig. 2, provide a comprehensive visualization of the observed patterns. A discernible trend emerges, indicating that the primary wind directions at the site predominantly originate from the southwest and northwest quadrants. Furthermore, the wind speeds exhibit a concentration within the 0–14 m/s range, characterized by varying levels and directions.

In studying the effect of wind on large radio telescope, Gawonski categorized the antenna wind load input models into three types: wind force acting directly on the main reflector surface of the antenna in the form of wind pressure, which causes deformation of the structure, equivalence of the wind force as a moment acting on the antenna drive shaft, and equivalence of the wind perturbation as an effect on the efficiency of the drive input. Among these types, the effect of wind pressure acting on the reflector surface is particularly pronounced [32].

Therefore, in this study, wind pressure prediction tests are conducted on the surface of the reflector at the KRT site in Kunming. This model enables real-time prediction of wind pressure, facilitating the development of wind resistance control strategies for the JRT to be installed at the Jingdong site.

## 2 Methodology

### 2.1 VMD algorithm

VMD is a self-adaptive, non-recursive modal variation and signal processing technique [33]. In comparison with other time-frequency decomposition methods, VMD demonstrates the capability to effectively reduce the non-smoothness of wind pressure time series, which are often characterized by high complexity and strong non-linearity. The specific expression for the variational constraint is as follows:

(1) $$\min_{\left\{u_{k}\},\{\omega_{k}\right\}}\left\{\sum_{k}{\left\|\partial_{t}[(\delta (t)+\frac{j}{\pi t})⁎u_{k}(t)]e^{-j\omega_{k}t}\right\|}_{2}^{2}\right\}s.t.\underset{k=1}{\sum^{K}}u_{k}=y(t)$$

Here, $u_{k}$ represents the finite bandwidth, $\omega_{k}$ (for k=1,2,...,K) signifies the center frequency, *K* denotes the number of Intrinsic Mode Functions (IMF_*S*_) to decompose, δ(t) represents the unit impulse function serving as the time indicator, and $\partial_{t}$ represents the partial derivative with respect to *t*. To solve variational constraints, it is necessary to introduce the Lagrange multiplier, and a quadratic penalty factor, denoted by *α*, is introduced to reduce the interference of Gaussian noise. The augmented Lagrange expression is represented by the following equation:

(2) $$L(\{u_{k}\},\{\omega_{k}\},\lambda )=\alpha \sum_{k}{\left\|\partial_{t}\left[(\delta (t)+\frac{j}{\pi t}⁎u_{k}(t)\right]e^{-i\omega_{k}t}\right\|}_{2}^{2}+{\left\|y(t)-\sum_{k}u_{k}(t)\right\|}_{2}^{2}+\left\{\lambda (t),y(t)-\sum_{k}u_{k}(t)\right\}$$

The alternating direction multiplier iterative algorithm is employed to solve the optimization problem, and the saddle point can be calculated, yielding the solutions of $u_{k}$ and $\omega_{k}$, and *λ* can be obtained.

(3) $$\begin{array}{c} \left\{ \begin{array}{cc} {\overset{ˆ}{u}}_{k}^{n+1}(\omega )\; & =\frac{\overset{ˆ}{y}(\omega )-\Sigma_{i\neq k}{\overset{ˆ}{u}}_{i}(\omega )+\overset{ˆ}{\lambda }(\omega )/2}{1+2\alpha {\left(\omega -\omega_{k}\right)}^{2}},\\ {\overset{ˆ}{\omega }}_{k}^{n+1}\; & =\frac{\int_{0}^{\infty }\omega |{\overset{ˆ}{u}}_{k}^{n+1}(\omega ){|}^{2},d\omega }{\int_{0}^{\infty }|{\overset{ˆ}{u}}_{k}^{n+1}(\omega ){|}^{2},d\omega },\\ {\overset{ˆ}{\lambda }}^{n+1}(\omega )\; & ={\overset{ˆ}{\lambda }}^{n}(\omega )+\tau (\overset{ˆ}{y}(\omega )-\sum_{k}{\overset{ˆ}{u}}_{k}^{n+1}(\omega )), \end{array}\right. \end{array}$$

where y(t) is the original signal, which is decomposed into *k* IMFs. *τ* represents the tolerance of noise. Additionally, ${\overset{ˆ}{u}}_{k}^{n+1}(\omega )$, ${\overset{ˆ}{u}}_{i}(\omega )$, $\overset{ˆ}{y}(\omega )$, and $\overset{ˆ}{\lambda }(\omega )$ are the Fourier transforms of $u_{k}^{n+1}(t)$, $u_{i}(t)$, y(t), and λ(t), respectively.

The alternating direction multiplier iterative algorithm is used to solve the optimization problem, the saddle point can be calculated, and the solutions of $u_{k},\omega_{k},\lambda$ can be obtained.

(4) $$\begin{array}{c} \left\{ \begin{array}{cc} {\overset{ˆ}{u}}_{k}^{n+1}(\omega )\; & =\frac{\overset{ˆ}{y}(\omega )-\Sigma_{i\neq k}{\overset{ˆ}{u}}_{i}(\omega )+\overset{ˆ}{\lambda }(\omega )/2}{1+2\alpha {\left(\omega -\omega_{k}\right)}^{2}},\\ {\overset{ˆ}{\omega }}_{k}^{n+1}\; & =\frac{\int_{0}^{\infty }\omega |{\overset{ˆ}{u}}_{k}^{n+1}(\omega ){|}^{2},d\omega }{\int_{0}^{\infty }|{\overset{ˆ}{u}}_{k}^{n+1}(\omega ){|}^{2},d\omega },\\ {\overset{ˆ}{\lambda }}^{n+1}(\omega )\; & ={\overset{ˆ}{\lambda }}^{n}(\omega )+\tau (\overset{ˆ}{y}(\omega )-\sum_{k}{\overset{ˆ}{u}}_{k}^{n+1}(\omega )), \end{array}\right. \end{array}$$

where y(t) is the original signal, it is decomposed into *k* IMFs, *τ* is the tolerance of noise and ${\overset{ˆ}{u}}_{k}^{n+1}(\omega )$, ${\overset{ˆ}{u}}_{i}(\omega )$, $\overset{ˆ}{y}(\omega )$, $\overset{ˆ}{\lambda }(\omega )$ are the Fourier transform of $u_{k}^{n+1}(t)$, $u_{i}(t)$, y(t), λ(t), respectively.

### 2.2 Time series prediction algorithms

While RNNs commonly employ a logistic nonlinear activation function for recurrent learning, their unique strength lies in their ability to capture time series dependencies in sequence data. This characteristic makes them particularly suitable for tasks in natural language processing, and they are widely used for solving time series prediction problems. However, when dealing with long series, RNNs face challenges related to the vanishing or exploding gradient problems. Specifically, in scenarios with extended time intervals, the gradient tends to approach zero or become excessively large. As a result, traditional recurrent neural networks encounter difficulties in effectively handling long-interval information sequences.

To address the issues of gradient vanishing and exploding, Long Short-term Memory (LSTM) [34] and Gated Recurrent Unit (GRU) [35] have been introduced as alternative RNN architectures capable of mitigating these challenges. LSTM incorporates a gating mechanism, including a forget gate ($f_{t}$), input gate ($i_{t}$), and output gate ($o_{t}$), aimed at mitigating the challenges faced by traditional recurrent neural networks. Similarly, GRU, with a simpler structure containing only two key gates (update gate and reset gate), possesses fewer parameters and enables faster training.

**Figure 3:**
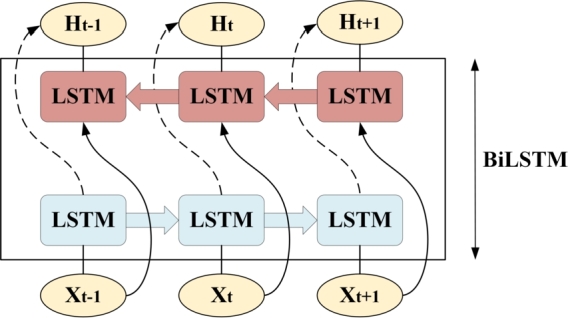
Basic structure of BiLSTM.

The Bidirectional Long Short-term Memory (BiLSTM) neural network comprises both forward and reverse LSTM models [36]. This architecture not only preserves the strengths of LSTM in effectively handling sequences with prolonged dependencies but also overcomes the limitation of insufficient data information in LSTM networks. The structure of the BiLSTM model is depicted in Fig. 3.

### 2.3 Evaluation metrics

To evaluate the prediction performance of various models, this study utilizes four statistical metrics: Root Mean Square Error (RMSE), Mean Absolute Error (MAE), Mean Absolute Percentage Error (MAPE), and the coefficient of determination ($R^{2}$). RMSE quantifies the disparity between the predicted and actual values of the model. MAE represents the average percentage difference between the predicted and actual values of the model. Similarly, MAPE denotes the average percentage deviation between the predicted and actual values of the model. Moreover, smaller values of RMSE, MAE, and MAPE indicate superior forecasting performance across various deep learning models. $R^{2}$ represents the goodness-of-fit of the model to the observed data. A value of $R^{2}$ close to one indicates outstanding performance of prediction models [37]. Below, the calculation method of each evaluation indicator is outlined.

(5) $$RMSE=\sqrt{\frac{1}{n}\overset{n}{\sum_{i=1}}(y_{i}-{\overset{ˆ}{y}}_{i})}$$

(6) $$MAE=\frac{1}{n}\overset{n}{\sum_{i=1}}|y_{i}-{\overset{ˆ}{y}}_{i}|$$

(7) $$MAPE=\frac{1}{n}\overset{n}{\sum_{i=1}}|\frac{y_{i}-{\overset{ˆ}{y}}_{i}}{y_{t}}|\times 100\text{\%}$$

(8) $$R^{2}=1-\overset{n}{\sum_{i=1}}\frac{{\left(y_{i}-{\overset{ˆ}{y}}_{i}\right)}^{2}}{{\left(y_{t}-\overset{¯}{y_{t}}\right)}^{2}}$$

## 3 Simulation setup

Finite Element Analysis (FEA) is the process of predicting the deflections and other stress effects on a structure, and Finite Element Modeling (FEM) divides this structure into a grid of cells to form a model of the actual structure. The displacement of the reflector surface is primarily influenced by the mean winds. By building a finite element model of the KRT reflector for analysis, the wind load distribution as well as the deformation on the reflector surface can be observed. References were made to the current wind load provisions in the codes for exposure roughness parameter estimation methods: the American Society of Civil Engineers Standard [38], the National Building Code of Canada [39], the Australian/New Zealand Standard [40], and the National Standard of the People's Republic of China for wind load provisions [41]. In this study, the wind load on the surface of the KRT reflector surface is calculated as follows:

(9) $$W=\beta_{z}\mu_{s}\mu_{z}\omega_{o}$$

where $\beta_{z}$ is the wind vibration coefficient, $\mu_{s}$ is the wind load body shape coefficient, $\mu_{z}$ is the wind pressure height change coefficient, $\omega_{o}$ is the basic wind pressure which is calculated as follows:

(10) $$\omega_{o}=\frac{1}{2}\rho_{air}{v_{b}}^{2}$$

In this study, we consider where $\rho_{\text{air}}$ represents the air density, and $v_{b}$ denotes the average wind speed at the standard reference height of 10 m. The wind vibration coefficient $\beta_{z}$ serves as an amplification factor to account for the randomness and dynamic characteristics of wind loads. In this paper, our focus lies on examining the effect of static force resulting from the average wind on the KRT reflector, thus $\beta_{z}$ is set to 1. The wind load form factor is determined using zonal average wind load form factor values, while the wind pressure height change factor is calculated using the following exponential equation:

(11) $$\mu_{z}=1.00\cdot {\left(\frac{Z}{10}\right)}^{2\alpha }$$

where Z represents the height above ground, the center of the reflector of the KRT is about 21.5 m above the ground, and the height of the reflector surface above the ground changes according to the working condition of the telescope. *α* is the ground roughness index, and α=0.15 for Class B terrain. The area where KRT is located is calculated according to Class B.

**Figure 4:**
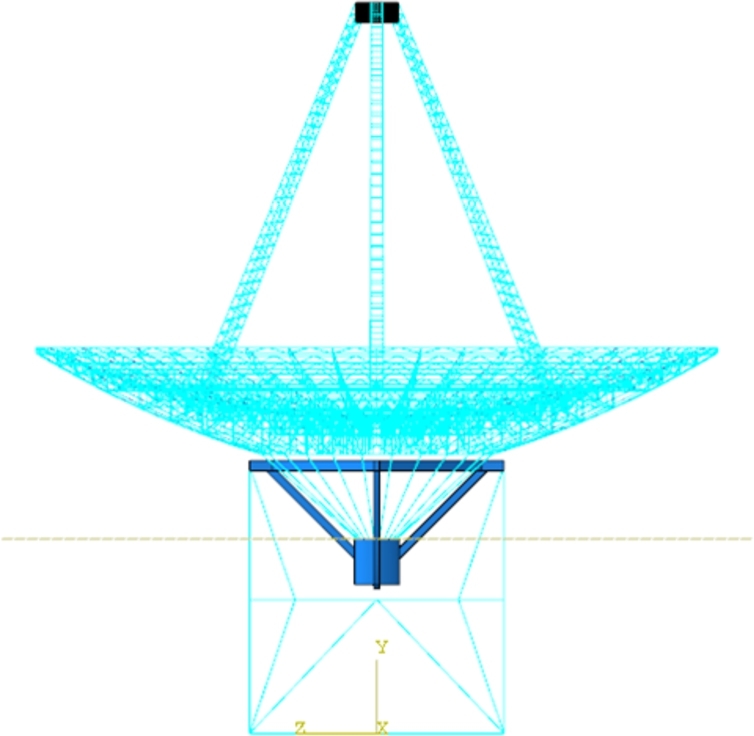
The FE model of the KRT at an pitch angle of 90°.

**Figure 5:**
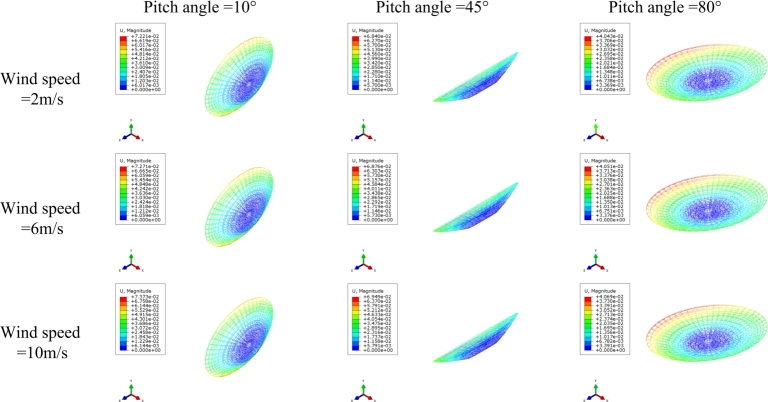
Reflector surface deformation under different operating conditions.

In the wind load calculation and application of the finite element model, the wind pressure is equated to the centralized wind load at each node of the reflector surface. The pressure coefficients of each reflecting surface panel are initially utilized in the telescope deformation analysis, surface error, and pointing error computations during wind loading processes. The wind forces at the nodes of the finite element model are determined by the products of accessory surface areas, wind pressures, and pressure coefficients [42]. The magnitude of the centralized wind load is calculated based on the node loading area and decomposed into three directions along the coordinate axes, thus completing the node load assignment. The finite element model of the KRT was constructed using the ABAQUS software, as illustrated in Fig. 4. The telescope structure comprises several components, including the reflector surface, pitching mechanism, seat frame, and feeder compartment. Certain components in the FE model are replaced by mass node elements to reflect real-world conditions. By varying wind speeds and adjusting the telescope's pitch angle, various wind loads can be generated and subsequently applied to the finite element model. The calculated wind loads were averaged over 1296 nodes on the surface of the reflecting surface to obtain the equivalent wind loads at each node on the reflecting surface. The gravity-induced deformation distribution varies with elevation angle and can be simulated by the antenna FE model. The change in the elevation angle represents that the gravity distribution of the entire antenna has changed. The smaller the pitch angle, the greater the impact of gravity. Although gravity loads can cause subtle deformation of the reflective surface, it is the effect of wind loads that is the dominant factor in the deformation of the reflective surface. The deformation of the reflector surface resulting from the FE analysis is depicted in Fig. 5. The calculated wind load is averaged over the 1296 nodes on the surface of the reflector surface to obtain the equivalent wind load on the reflective surface. Although gravity loads can cause subtle deformation of the reflective surface, it is the effect of wind loads that is the dominant factor in the deformation of the reflective surface. With the same wind speed, a smaller pitch angle on the reflector surface leads to more severe deformation. Similarly, with the same pitch angle, a higher wind speed leads to greater deformation on the surface, the more wind disturbance affects the pointing accuracy and face shape accuracy. In conditions of high wind speed, with a minimum pitch angle of 10° and a wind speed of 10 m/s, the maximum combined displacement on the reflecting surface reaches 72.73 mm. Conversely, under low wind speed conditions, with a pitch angle of 80° and a wind speed of 2 m/s, the maximum combined displacement is reduced to just 40.43 mm. Different wind loads lead to varying displacements of the reflector surface across different positions, highlighting the need to partition reflector surface panels for analyzing wind pressure distribution.

## 4 Wind pressure data acquisition

**Figure 6:**
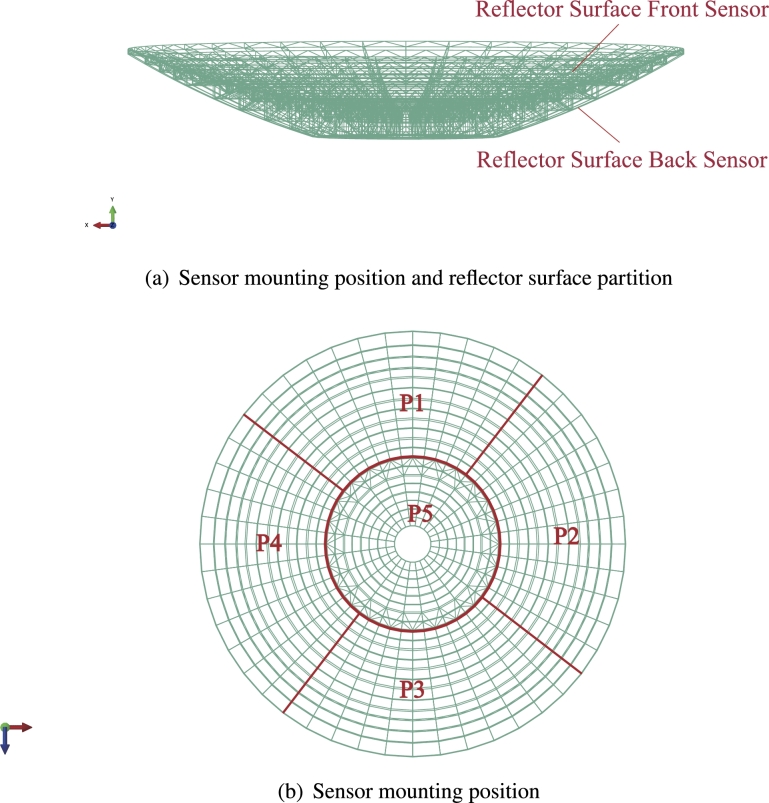
Reflector surface partition.

In this study, we conducted a case study utilizing wind data from the KRT site. Wind loads were calculated by directly measuring the wind pressure on the telescope's reflector surface. This approach allows for the consideration of wind loads regardless of the specific direction of incoming winds. The reflector surface is segmented into five large areas, each corresponding to the actual installation positions of five groups of differential pressure sensors. Additionally, the reflector is subdivided into 20 rings, 8 on the inner side and 12 on the outer side, providing a detailed spatial breakdown for precise analysis. Real-time wind pressure data were collected using five sets of micro differential pressure sensors mounted on the reflector surface of the telescope. The specific mounting positions of the sensors is shown in Fig. 6(a), both front and back, the partitioning of the reflector surface is shown in Fig. 6(b).

**Figure 7:**
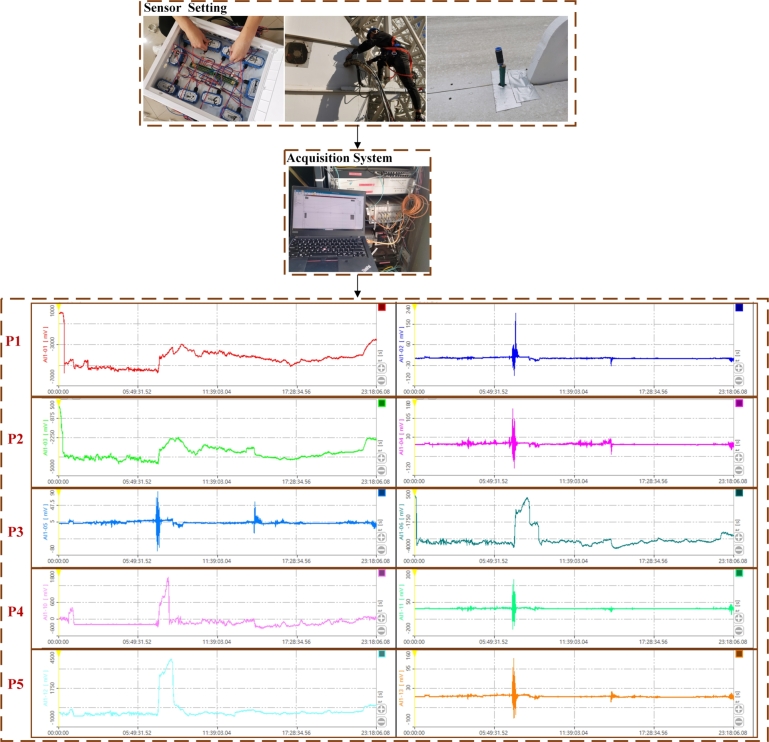
Flowchart of wind pressure data acquisition.

The sensors utilized are SETRA-268 pressure sensors, strategically placed in five partitions on the telescope surface. Each partition is subdivided into two ports, enabling the measurement of pressure differences between the front and back surface areas. The wind pressure sensor is connected via an air conduit with an approximate diameter of 3 mm. The wind pressure captured by the conduit on the reflecting surface creates a pressure difference with the room's atmospheric pressure, acting directly on the sensor's diaphragm. This interaction produces a micro-displacement proportional to the medium's pressure. This displacement alters the sensor's resistance, which is detected by an electronic circuit and converted into a standard signal corresponding to the pressure. The Dynamic Signal Harvesting and Analysis System (DSHAS) is employed to capture and display the changes in these voltage values. The conversion relationship between the pressure value and the voltage change value collected by the sensor is shown in Table 1. This system captures and displays the changes in voltage values, which correspond to the pressure differences measured by the sensors. Fig. 7 depicts the field collection of wind pressure data and the collection interface of DSHAS. Each row in the figure represents the position of a sensor pair, with the left side displaying data from sensors mounted on the front side of the reflector surface, while the right side displays data from sensors mounted on the opposite side.

**Table 1:** Calibration chart.

Applied Pressure (PASCAL)	Transducer Output (VDC)
-980.3109	0.0979
-791.5609	0.5657
-594.6234	1.0565
-397.8499	1.5486
-170.9515	2.1166
14.4470	2.5836
201.2673	3.0537
417.5173	3.5967
604.7595	4.0676
800.8376	4.5566
1032.2750	5.1311

**Figure 8:**
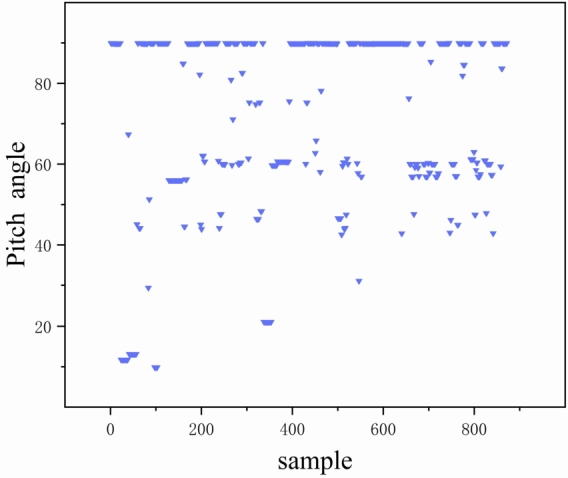
Steady state working condition distribution.

The sensor is set to collect at 50 Hz, and the raw data collected needs to be aligned under the same time. To ensure the analysis is conducted under stable operating conditions and mitigate the impact of telescope motor operations, the data is filtered. Specifically, data is analyzed during steady operating conditions, where changes in pitch attitude are less than 2°. The steady-state data is then aggregated into 10-minute intervals for comprehensive analysis. In Fig. 8, it is evident that pitch angles of 10°, 45°, 60°, and 90° are more prevalent in all the screened smooth time states. These pitch angles cover a broad range of wind conditions and will be the focus of our wind pressure analyses and predictions.

Analyzing the distribution characteristics of mean and fluctuating wind pressures on large telescope mirrors is crucial for their wind-resistant design. In particular, the average wind pressure reflects the impacts received by the reflector surface over a period of time. The average wind speed is the basis for calculating the wind load, as the reflector surface is subjected to wind pressure that changes with the wind speed. Thus, the calculation of wind load can be simplified by directly obtaining the average wind pressure. Therefore, this study aims to simplify the calculation of wind loads by measuring the wind pressure directly on the reflector surface. After selecting the data over a one-hour period, the raw wind pressure data collected can be averaged over multiple points to obtain the average change in wind pressure per minute on the reflector surface. This can be a more intuitive reflection of the impact of wind disturbances per minute. Subsequently, the proposed hybrid prediction model is trained based on 80% of the data, and its prediction performance is evaluated using the remaining 20% of the data.

## 5 Framework of the proposed model

Based on the outlined methodology, we have developed an efficient wind pressure prediction model. The model prediction flow comprises five components: VMD decomposition, normalization, model training, model prediction, and performance evaluation. The primary steps of the proposed model are explained below:

(i) Wind pressure data decomposition

**Figure 9:**
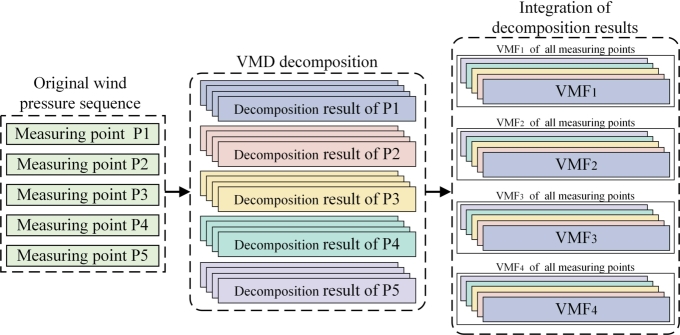
Wind pressure decomposition.

Decompose the original wind pressure sequence using VMD to obtain the distinct Variational Mode Function (VMF) components. The decomposition process is illustrated in Fig. 9, where VMF_*i*_ (i=1,2,3,4) denotes the outcomes of the VMD decomposition. Once the appropriate number of modes *k* and penalty parameter *α* are determined, the sequences can be decomposed into four components using the VMD method. The data from each measurement point are decomposed individually and then integrated according to the component order, resulting in a new set of four integrated datasets. These decomposed wind pressure components exhibit distinct features, and this feature extraction aids in wind pressure prediction.

(ii) Model training and prediction

**Figure 10:**
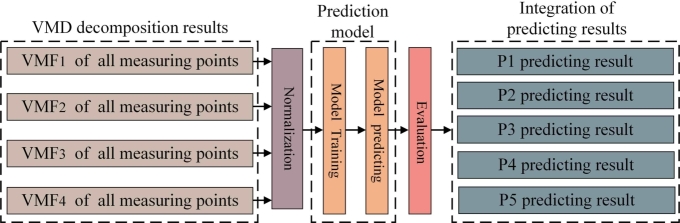
Model training and prediction.

As shown in Fig. 10, after the VMD decomposition algorithm, it will be reintegrated into new sequences, each containing the results of different measurement points under the same VMF component. First, these sequences will be normalized using the min-max method. Then, prediction models are trained and their performance evaluated. Finally, the predictions will be reconstructed to the original scale and outputted.

(iii) Performance evaluation

Four prediction performance evaluation metrics, namely $R^{2}$, RMSE, MAE, and MAPE, were chosen to assess and compare the performance of the proposed predictive model against other models. RMSE, MAE, and MAPE represent calculations of sample point errors, while $R^{2}$ quantifies the fitting effect between predicted and actual values. This study processed and predicted data from various smooth state pitch conditions to synthesize and compare model performance.

## 6 Prediction results and discussion

**Figure 11:**
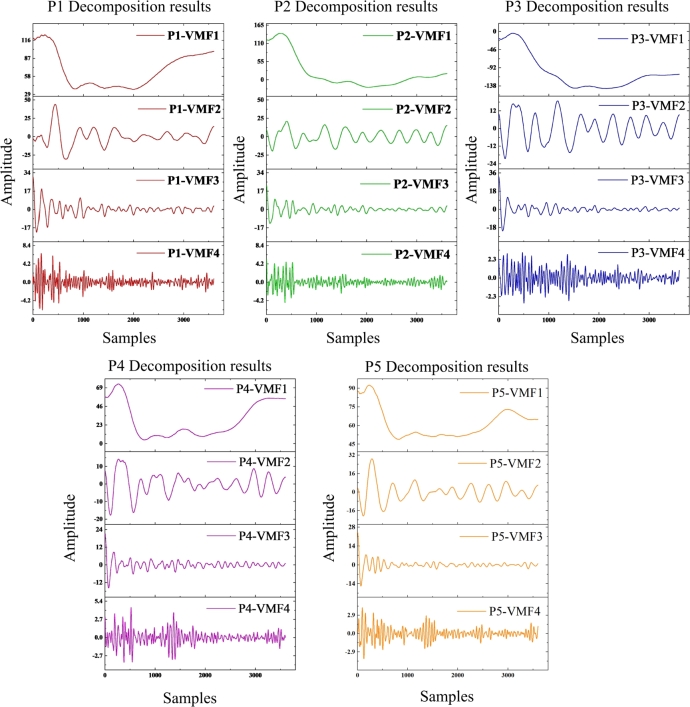
Results of VMD decomposition for all measurement points.

This section compares the performance of six different models based on the VMD method, encompassing both single-model prediction approaches and prediction models incorporating VMD decomposition. The models included in the comparison are LSTM, GRU, and BiLSTM, as well as their respective counterparts integrated with the VMD decomposition method. After following the decomposition process outlined in the methodology, the wind pressure series is decomposed into four VMF components. Fig. 11 depicts the wind pressure data after decomposition under smooth conditions with a pitch angle of 45°. The VMD decomposition of the wind pressure signals reveals that while the overall trends are similar across the sensors, there are subtle differences in the oscillatory components at different frequencies. In each of the five sets of wind pressure sequence plots, the original sequence and the decomposed four-component sequence are shown. The first VMF component captures the low-frequency components of the wind pressure signal collected. The curve shows a smooth variation, reflecting the long-term trend in the pressure signal. The second and third VMF components exhibit mid-frequency oscillations with a small amplitude. The last component contains significant high-frequency component, indicating the presence of high-frequency noise or minor random fluctuations in the wind pressure signal.

Table 2 displays the configuration parameters of the prediction models. All models are configured with identical settings for hidden layers, epochs, learning rate, optimizer, batch size, and dropout rate, facilitating equitable comparison of algorithm performance. To select the best parameters, we initially set a range of possible values for the learning rate, batch size, and other hyperparameters. During the grid search, the Adam optimizer iteratively adjusted the parameters within this range to minimize the loss function. The combination of parameters that resulted in the lowest validation loss was chosen as the optimal set. We used the Adam optimizer with a learning rate of 0.001. Models were trained on 1000 epochs with a batch size of 32.

**Table 2:** The setting parameters for prediction models.

Parameters	LSTM	GRU	BiLSTM
Depth	3	3	3
Hidden	[32,32,16,1]	[32,32,16,1]	[32,32,16,1]
Epochs	1000	1000	1000
Learning rate	0.001	0.001	0.001
Optimizer	Adam	Adam	Adam
Batch size	32	32	32
Dropout	0.2	0.2	0.2

Following the decomposition process, the data are grouped into sets of VMF components. Each group of integrated data encapsulates the features of the respective component at various measurement points. These integrated data sets are then fed into the prediction model using a multivariate input approach, accounting for both the temporal and spatial correlation of the wind pressure data on the reflector surface. VMD simplifies the representation of complex high-frequency components into multiple subsequences and ensures effective decomposition with fewer components. This approach enhances the model's ability to capture the underlying patterns in the wind pressure data.

For the proposed model, 80% of the data set is used for training, that is, the first 48 minutes. Under these conditions, the proposed model demonstrates good performance in short-term wind pressure prediction. In order to evaluate its effectiveness, a single model and two hybrid models are compared with the proposed model. The model performance is evaluated by calculating the error and goodness of fit of each corresponding model.

As indicated in Table 3, the prediction performance of BiLSTM surpasses that of LSTM and GRU without modal decomposition. Comparing individual models (LSTM, GRU, and BiLSTM), the proposed model achieves significantly smaller MAE, RMSE, and MAPE. The proposed hybrid model demonstrates the best performance with an average $R^{2}$ of 0.9392, and the values of RMSE, MAE, and MAPE are 1.4923, 1.2377, and 1.82%, respectively. Additionally, prediction models with modal decomposition outperform single models. Notably, the proposed model exhibits superior performance when VMD decomposition is applied. The assessment of predicted results under different operating conditions is presented in Table 4. Particularly, under commonly occurring 90° conditions, $R^{2}$ reaches 0.9705, indicating the high accuracy of the proposed model.

**Table 3:** Comparison of prediction performance between proposed model and other models at pitch angle of 45.

Model	*R*^2^	RMSE	MAE	MAPE
LSTM	0.4577	4.7638	3.7017	5.24%
GRU	0.7178	3.4713	2.9669	3.30%
BiLSTM	0.7895	2.6807	2.2806	2.64%
VMD-LSTM	0.9144	1.7390	1.4346	2.14%
VMD-GRU	0.9319	1.6623	1.3739	1.97%
Proposed model	0.9392	1.4923	1.2377	1.82%

**Table 4:** Performance of all reflector surface measuring points at all working conditions.

Pitch angle	Models	*R*^2^	RMSE	MAE	MAPE
10	VMD-LSTM	0.6714	8.2323	6.9898	4.52%
	VMD-GRU	0.8058	6.4171	5.4505	5.64%
	Proposed model	0.8060	6.3210	5.3967	3.92%

45	VMD-LSTM	0.9144	1.7390	1.4346	2.14%
	VMD-GRU	0.9319	1.6623	1.3739	1.97%
	Proposed model	0.9392	1.4923	1.2377	1.82%

60	VMD-LSTM	0.7590	8.5440	6.8944	2.53%
	VMD-GRU	0.7479	8.6388	7.1445	2.80%
	Proposed model	0.8312	7.1032	6.0743	2.41%

90	VMD-LSTM	0.9541	1.6410	1.2842	0.82%
	VMD-GRU	0.9595	1.5557	1.2288	0.76%
	Proposed model	0.9705	1.3153	1.0355	0.61%

**Figure 12:**
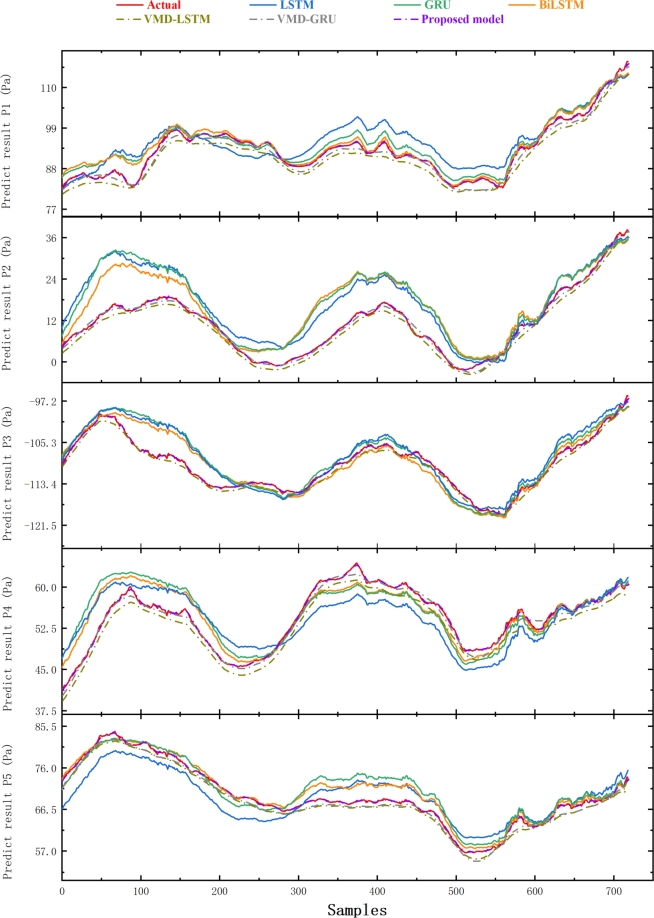
Comparison of prediction results with other models.

**Figure 13:**
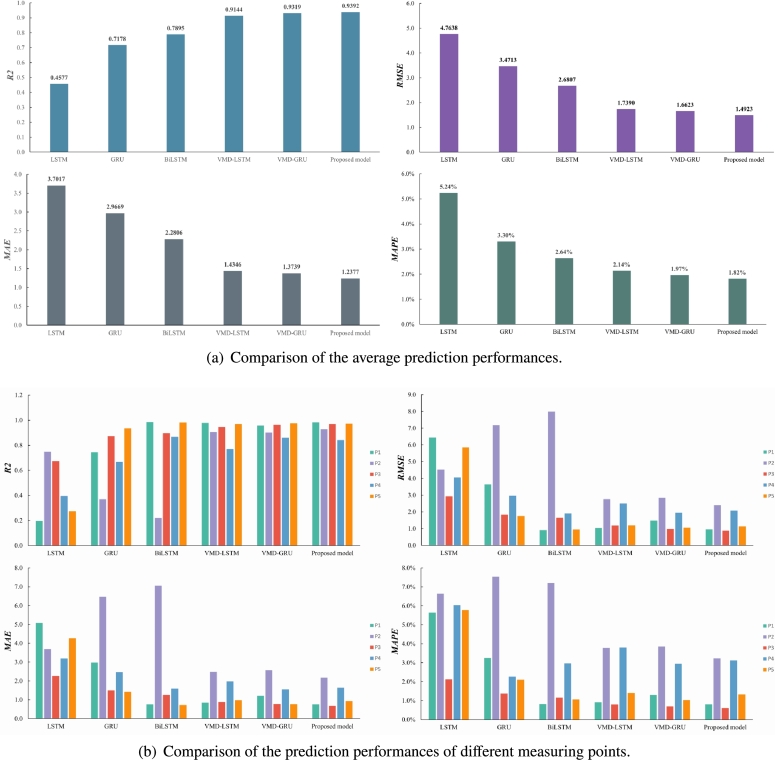
Comparison of the performance of each measurement point and the overall average prediction performance.

The actual wind pressure values and the predicted values of the proposed model and other models are depicted in Fig. 12. The decomposition-based model generally follows the trend of the actual value curve, albeit with some deviation points. In contrast, the proposed model closely aligns with the actual value curve, demonstrating excellent predictive performance. Fig. 13(a) depicts the Comparison of the average prediction performances of models on different data sets. Fig. 13(b) depicts the comparison of the prediction performances of different measuring points. The single model provides a poor fit to the prediction results at measurement point P2. However, the prediction method combined with modal decomposition yields more balanced predictions and better overall performance. The proposed model exhibits the smallest MAE, RRMSE, MAPE, and the highest $R^{2}$ across different data sets. Moreover, the prediction curve of this model is closest to the true value among the six prediction models, indicating good and stable robustness in predicting the reflector surface average wind pressure series. The feature extraction of wind pressure trends by the VMD algorithm enhances the prediction model's ability to capture the wind pressure trend and distribution characteristics, resulting in improved prediction accuracy.

## 7 Conclusions

In this study, we employed a combination of field-measured data and deep learning to accurately decompose and predict the wind pressure on the reflector surface of a large-aperture radio telescope. Introduced is a reflector surface wind pressure prediction multi-scale model based on VMD decomposition and prediction models, which mitigates non-smoothness in complex and nonlinear wind pressure time series. The BiLSTM model, incorporating cell states and a gate structure for information control, effectively captures long-term dependencies in time series data. This paper addresses the challenge of optimizing the performance of radio telescopes in windy environments, particularly in accurately estimating wind pressure on the reflector surface. The proposed approach aims to ensure that the telescope can maintain stable and efficient operation under various meteorological conditions. The integration of time series prediction with deep learning demonstrates significant potential in the design and optimization of radio telescopes, especially in handling the effects of wind fields in complex environments. The proposed method offers a novel and effective solution to the wind disturbance problem faced by large radio telescopes. Future work will increase the types of independent variables to get more scientific predictions of specific deformations of telescope reflector surfaces by considering the link between reflector surface displacement and wind pressure.

## CRediT authorship contribution statement

**Rui Wu:** Writing – original draft, Visualization, Software, Methodology, Data curation. **Zhong Cao:** Writing – review & editing, Writing – original draft, Supervision, Project administration, Methodology, Conceptualization. **Feng Wang:** Writing – review & editing, Supervision, Methodology, Funding acquisition. **Rui Rao:** Writing – original draft, Validation, Methodology, Data curation. **Yuxiang Huang:** Writing – original draft, Validation, Data curation. **Ruifeng Hu:** Writing – original draft, Visualization, Software, Data curation.

## Declaration of Competing Interest

The authors declare that they have no known competing financial interests or personal relationships that could have appeared to influence the work reported in this paper.

## Data Availability

Data will be available from the author on reasonable request.
